# Associations of Plasma Erythritol with Dietary Factors, Cardiometabolic, Inflammatory, and Gut Health Markers in People with and without HIV: A Cross-Sectional Study

**DOI:** 10.3390/nu16203449

**Published:** 2024-10-11

**Authors:** Aaron A. Fletcher, Jared C. Durieux, Ilya Bederman, John Feczko, Ornina Atieh, Jhony Baissary, Danielle Labbato, Kate Ailstock, Nicholas T. Funderburg, Grace A. McComsey

**Affiliations:** 1 School of Medicine, Case Western Reserve University, 10900 Euclid Ave., Cleveland, OH 44106, USA; irb2@case.edu (I.B.); jpf85@case.edu (J.F.); ornina.atieh@case.edu (O.A.); jhony.baissary@case.edu (J.B.); grace.mccomsey@uhhospitals.org (G.A.M.); 2 Center for Clinical Research, University Hospitals Cleveland Medical Center, Cleveland, OH 44106, USA; jared.durieux@uhhospitals.org (J.C.D.); danielle.labbato@uhhospitals.org (D.L.); 3 Division of Medical Laboratory Science, School of Health and Rehabilitation Sciences, The Ohio State University, 453 W. 10th Ave., Columbus, OH 43210, USA; kate.ailstock@osumc.edu (K.A.); nicholas.funderburg@osumc.edu (N.T.F.)

**Keywords:** erythritol, HIV, major adverse cardiovascular events (MACE), pentose phosphate pathway (PPP)

## Abstract

Background: Recently, elevated levels of plasma erythritol have been associated with major adverse cardiovascular events (MACE). It is known that people with HIV (PWH) have a higher cardiovascular disease burden. Whether PWH have higher levels of plasma erythritol has not been evaluated. This study aimed to assess if blood erythritol levels are elevated in PWH and to examine relationships between erythritol and dietary, cardiometabolic, inflammatory, and gut health markers. Methods: Plasma erythritol levels were measured using frozen samples from 162 participants, including 109 PWH and 53 people without HIV (PWoH) in a parent study. General linear models were used to assess the linear relationship between characteristics, cardiovascular measures, markers of body composition, inflammation, and gut integrity with plasma erythritol. Logistic regression was used to assess risk factors associated with PWH, and cumulative logit models were used to investigate which factors were associated with having the highest plasma erythritol levels among PWH. Results: Compared to PWoH, PWH had higher plasma erythritol levels (*p* = 0.03). Every 10% increase in VLDL (*p* = 0.01), visceral adipose tissue (*p* < 0.0001), or TNFrI (*p* = 0.01) was associated with an approximately 1% increase in plasma erythritol. Among PWH, HgbA1c (*p* = 0.003), TNFrI (*p* = 0.002), and IFAB-P (*p* = 0.004) were associated with having the highest tertile of plasma erythritol (≥3.6 μM). Compared to PWoH, PWH were more than two times as likely (*p* = 0.03) to have plasma erythritol ≥ 3.6 μM. Conclusions: We identified positive associations between plasma erythritol levels and several factors, including HIV status, BMI, adipose tissue, TNFr1, HbA1c, and VLDL. These results underscore the importance of further investigating the role of elevated plasma erythritol levels in people with HIV, particularly in light of their increased vulnerability to cardiovascular and metabolic diseases.

## 1. Introduction

Chronic, non-communicable diseases account for significant morbidity and mortality in people with HIV (PWH). Specifically, PWH have a significantly higher cardiovascular disease burden when compared to people without HIV (PWoH). Witkowski et al. recently found erythritol to be highly correlated with incident 3-year risk for major adverse cardiovascular events (MACE; includes death or non-fatal myocardial infarction or stroke) [1]. These findings are consistent with a prior work that found erythritol to be positively associated with increased cardiovascular risk [2]. Historically, it was thought that erythritol was sourced exclusively from the diet; however, more recently, it was found to be synthesized endogenously from glucose via the pentose phosphate pathway (PPP) in stable isotope-assisted ex vivo blood incubation experiments and through the in vivo conversion of erythritol to erythronate in stable isotope-assisted dried blood spot experiments [3].

In recent studies, HIV infection was associated with metabolic adaptations, including increased activity of the PPP, as evidenced by the increased presence of PPP products, ribose-5-phosphate, and erythrose-4-phosphate [4]. Despite antiretroviral therapy (ART) initiation, the PPP remained abnormally regulated, despite viral suppression [4]. It is theorized that HIV itself may upregulate PPP to produce products necessary to support infection and antioxidant capacity to combat host response-induced oxidative stress by generating both ribose-5-phoshpate and NADPH, respectively [4,5]. Ortiz et al. demonstrated that erythritol synthesis is modulated by both glucose availability in a dose-dependent manner and oxidative stress in human lung carcinoma cells [6]. They suggested that the co-occurrence of hyperglycemia and oxidative stress may be a crucial factor in increasing erythritol levels. In a separate study, Ortiz et al. determined that sucrose intake, not high-fat diet, increased erythritol synthesis and excretion in mice [7]. Glycemic derangements in PWH are well documented; however, whether those abnormalities are associated with elevated erythritol levels in PWH has yet to be elucidated.

Another metabolic disturbance related to HIV infection, visceral adipose tissue (VAT) accumulation, has been associated with systemic and adipose tissue inflammation, increased oxidative stress, dyslipidemia, and insulin resistance [8,9,10,11]. Hootman et al. found an association between incident central fat gain and higher blood erythritol levels in college participants [3].

Considering the associations between blood erythritol levels and MACE, central adiposity, and pentose phosphate pathway derangements associated with HIV infection, we aimed to determine whether blood erythritol levels are elevated in PWH, in addition to discerning whether relationships exist between erythritol levels and dietary factors, VAT, inflammatory markers, and cardiovascular risk markers to better help understand the chronic, non-communicable ailments disproportionately afflicting this population.

## 2. Materials and Methods

### 2.1. Study Design

Control samples were sourced from participants in a parent study and were HIV-negative (people without HIV (PWoH)) and ≥18 years old, had a BMI ≥ 25 kg/m^2^, and had a waist circumference and waist-to-hip ratio > 95 cm and >0.94, respectively, for men, and >94 cm and >0.88, respectively, for women. All evaluations and blood were collected and processed in a similar way for controls as for PWH. This paper reports on the baseline time point of all participants of the parent study.

Experimental frozen plasma samples were sourced from PWH participants in a parent study with the following inclusion criteria: participants were ≥18 years old, with documented HIV-1 infection and cumulative ART duration ≥ 1 year, receiving a stable ART regimen for ≥12 weeks and HIV-1 RNA < 400 copies/mL for ≥6 months prior to entry, with no plans to alter or stop their ART regimen during the study period. In addition, participants required subjective observation of increased abdominal girth occurring after ART initiation, a waist circumference (WC) and waist-to-hip ratio (WHR) of >95 cm and >0.94 cm, respectively, for men, and >94 cm and >0.88 cm, respectively, for women, and a body mass index (BMI) of ≥25 kg/m^2^.

The main exclusionary criteria were as follows: diabetes, history of MACE, acute or uncontrolled chronic inflammatory condition, infection, or cancer; excessive alcohol use (average ≥ 2 drinks daily); pregnancy or breastfeeding; and weight > 205 kg, since the latter is the limit of weight acceptable for our CT scanner machine.

### 2.2. Measurement of Biomarkers

Blood was collected by venipuncture, and plasma was isolated by centrifugation and was stored at −80 °C until processing as a batch without a prior thaw. Markers of systemic inflammation were measured using enzyme-linked immunosorbent assay (ELISA). The markers of interest and their respective manufacturer were the following: soluble CD14 (Cat. No. DC140) and CD163 (Cat. No. DC1630), high-sensitivity C-reactive protein (hsCRP) (Cat. No. DCRP00), interleukin-6 (IL-6) (Cat. No. HS600B), soluble receptors of tumor necrosis factor α (sTNFR-I and sTNFR-II) (Cat. Nos. DRT100 and DRT 200, respectively), interferon γ-inducible protein 10 (IP-10) (Cat. No. DIP100), the cellular adhesion molecules soluble vascular cell adhesion molecule 1 (sVCAM-1) (Cat. No. DVCOO), soluble intercellular adhesion molecule 1 (sICAM-1) (R&D Systems, Minneapolis, MN, USA; Cat. No. DCD540), D-dimer (Diagnostica Stago, Parsippany, NJ, USA; Cat. No. NC9884012), and oxidized low-density lipoprotein assays (Mercodia, Uppsala, Sweden; Cat. No. NC9664711). Several markers of gut health were also measured: zonulin (Promocell, Heidelberg, Germany; Cat. No. 50-311-083), intestinal fatty acid-binding protein (I-FABP) (R&D Systems, Minneapolis, MN, USA; Cat. No. DFBP20), lipopolysaccharide-binding protein (LBP) (Hycult Biotech, Plymouth Meeting, PA, USA; Cat. No. HK315-02), and (1 → 3)-β-D-Glucan (BDG) (Mybiosource Inc., San Diego, CA, USA; Cat. No. MBS756415) were measured by enzyme-linked immunosorbent assays (ELISA).

### 2.3. Assessment of Cardiovascular Disease Markers

An indirect evaluation of endothelial function was performed non-invasively using post-occlusive reactive hyperemia peripheral arterial tonometry (RH-PAT) (EndoPAT 2000 device; Itamar Medical Ltd., Atlanta, GA, USA), as we previously detailed [DOI: 10.3791/2167]. The reactive hyperemic index (RHI) (normal is >1.67) was generated. Additionally, the augmentation index (AI) was calculated, and the result was normalized to the heart rate of 75 beats per minute. Lower AI values reflected better arterial elasticity.

### 2.4. Determination of Plasma Erythritol Concentrations

Samples were processed using BSL2 precautions. De-identified plasma samples were thawed on ice, and a 50 μL aliquot was spiked with 10 μL of 0.01 mg/mL internal standard ([U-^13^C]glucose) for quantification. Erythritol was extracted by the addition of 1 mL of ice-cold acetone and vortexed for 1 min. Samples were incubated on ice for 30 min and vortexed again. Next, samples were centrifuged (14,000 rpm, 4 °C, 10 min), and supernatants were transferred to GC/MS vials. Extraction with acetone was repeated once more, and combined supernatants were evaporated to dryness. Erythritol and glucose standard were acetylated by the addition of 150 μL of pyridine acetyl anhydride (1:2) mix at 70 °C for 60 min. Derivatized samples were then evaporated to dryness and reconstituted in 60 μL of ethyl acetate and transferred to inserts for GC/MS analysis.

### 2.5. GC/MS Analysis

Erythritol and glucose standard were quantified using an Agilent 5977C MSD equipped with an Agilent 7890C GC system (GC/MS). A DB17-MS capillary column (60 m × 0.25 mm × 0.25 μm) was used in all assays with a helium flow of 1.5 mL/min. Gas chromatography was as follows: a 1 µL sample was injected in splitless mode; the injection was held at 270 °C; and the oven temperature was started at 150 °C and held for 1 min, then ramped up at 15 °C/min until it was 260 °C and held for 5 min, then ramped up at 30 °C/min until it was 300 °C and held for 5.3 min isothermally. The detector was held at 280 °C isothermally. A mass spectrometer was operated for electron impact ionization (EI), and ions were collected in SIM (selected ion monitoring) mode. Ion dwell time was set to 10 ms. Erythritol tetra-acetate ion *m*/*z* 217 was collected and quantified using an internal standard ion of *m*/*z* 205. Ion abundances were converted to relative concentrations (μM, or nmols/mL) using the standard curve.

### 2.6. Biochemical Analysis

In the parent study, participants underwent comprehensive clinical and laboratory assessments, including a comprehensive metabolic panel and lipoprotein profile. A twelve-hour fasting period was required prior to the blood draw. Glycated hemoglobin (HbA1c%) and glucose (fasting and 2 h after the consumption of 75 mg of oral glucose) were measured. HIV-1 RNA and CD4+ T-cell counts were obtained as part of the standard of care. Dietary assessments were based on 24 h food and supplement intakes (obtained by an experienced nutrition research core overseen by a registered dietitian). Data were analyzed using the Nutrition Data System for Research software (version 2018). One 24 h food or supplement recall was obtained at the participants’ study visits; phone calls were placed on subsequent days to obtain a total of three 24 h food and supplement recalls per time point.

### 2.7. Body Composition Analysis

Body composition was assessed using whole-body dual-energy X-ray absorptiometry (DXA) and non-contrast helical computed tomography (CT) of the chest and abdomen. All measurements were read by a single radiologist masked to the treatment assignment and clinical information. DXA was performed with a standardized protocol in anteroposterior view using the same scanner (Lunar Prodigy Advance, GE Healthcare, Chicago, IL, USA) for fat (total body, limb, and trunk) and lean body mass (LBM) measurements. Abdominal CT was performed with 3 mm scan increments extending from the dome of the diaphragm through the symphysis pubis. A single slice at the L4–L5 level was used to estimate abdominal adipose tissue (AT) quantity (area in cm^2^) delineated by compartment (VAT and SAT) and combined total (TAT).

### 2.8. Statistical Analysis

Continuous variables were described using mean ± standard deviation or median and interquartile range (IQR). Categorical variables were described using numbers (*n*) and percentages (%). Differences in baseline characteristics, including dietary intake between PWH and PWoH, were computed using either independent *t*-test, Wilcoxon Mann–Whitney U, or chi-square. General linear models were used to assess the linear relationship between characteristics, cardiovascular measures, markers of body composition, inflammation, and gut integrity with plasma erythritol. Logistic regression was used to assess risk factors associated with PWH, and cumulative logit models were used to investigate which factors were associated with having the highest plasma erythritol levels among PWH. The variance inflation factor was used to assess intercorrelation among independent variables, and a chi-square score test was used to test the proportional odds assumption. Adjusted models included HIV status, sex, race, age, and BMI as offset variables. Log transformations were used to reduce error variance and were interpreted as the percent change in linear models. All analyses were conducted using SAS 9.4 (SAS Inc., Cary, NC, USA), and *p*-values less than alpha < 0.05 were considered statistically significant.

## 3. Results

Baseline characteristics: A total of 162 participants were included in this study (Table 1). Among PWH (n = 109), 42.2% were females, 55.1% were of a non-white race, the mean viral load was 33.1 ± 60.2 copies/mL, and the absolute CD4 count was 833.6 ± 428.6 cells/mm^3^. Compared to PWoH, PWH were older [PWH 49.2 vs. (PWoH) 44.1 years; *p* = 0.01] and had a higher median BMI [(PWH) 33.6 kg/m^2^ vs. (PWoH) 29.4 kg/m^2^; *p* = 0.0002]. Markers of endothelial function (RHI) and arterial stiffness (AI) were similar (*p* > 0.05) between the two groups.

In Table 2, the median total calories among PWH was 1712.9 kcal, total fat was 69.8 g, total carbohydrates was 186.2 g, total sugars was 76.7 g, total protein was 63.7 g, and the average dietary erythritol was 0.02 g. Compared to PWoH, PWH reported 11.7 g of total saturated fat [vs. PWoH (14.5 g); *p* = 0.02], 12.8 g of monounsaturated fat [vs. PWoH (15.5 g); *p* = 0.04], 10.7 g of total fiber [vs. PWoH (13.7); *p* = 0.05], and 3.7 g of soluble fiber [vs. PWoH (5 g); *p* = 0.02]. Overall, the dietary intakes of carbohydrates, sugars, total fiber, and erythritol were similar between groups.

Among PWH, 45.9% (n = 50) had plasma erythritol > 3.6 μM, 26.6% had <2.7 μM, and the median plasma erythritol was 3.5 μM (IQR: 2.7, 4.5). Among PWoH, 33.9% (n = 18) had erythritol > 3.7 μM, 45.3% had <2.7 μM, and the median erythritol was 3.0 μM (IQR: 2.2, 4.1). The estimated difference in plasma erythritol between PWH and PWoH was 12.2% (*p* = 0.03; Table 3). In adjusted models, every 10% increase in VLDL (*p* = 0.01), visceral adipose tissue (*p* < 0.0001), or TNFrI (*p* = 0.01) was associated with an approximately 1% increase in plasma erythritol. In adjusted models, HIV status (*p* = 0.001), BMI (*p* = 0.001), VLDL (*p* = 0.01), visceral adipose tissue (*p* < 0.0001), and TNFrI (*p* = 0.01) remained positively associated with plasma erythritol. There was not enough evidence to suggest that total dietary fiber, soluble fiber, or dietary fiber was statistically associated with plasma erythritol.

**Figure 1:**
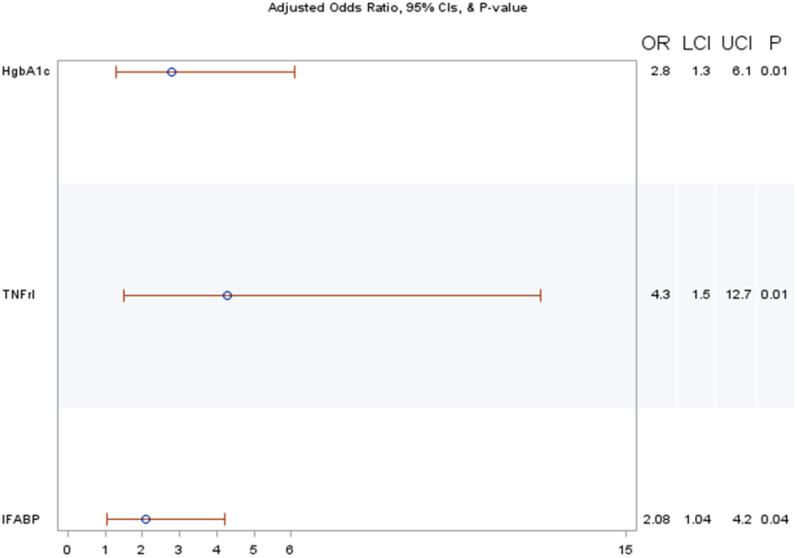
Risk factors associated with the highest plasma erythritol concentrations (≥3.6 μM) in PWH. Abbreviations: OR = odds ratio; LCI = lower confidence interval; UCI = upper confidence interval.

Among PWH (Figure 1), every unit increase in HgbA1c (*p* = 0.003), TNFrI (*p* = 0.002), and IFAB-P (*p* = 0.004) was associated with having the highest (≥3.6 μM) plasma erythritol.

In Table 4, compared to PWoH, having plasma erythritol > 3.6 μM is 2.3 times [(95% CI: 1.1, 4.9); *p* = 0.03] more likely to be associated with PWH. Similarly, when considering plasma erythritol as a continuous variable, every unit increase in erythritol increased the likelihood of being associated with PWH more than two-fold (*p* = 0.03). In adjusted models, every unit increase in plasma erythritol, subcutaneous adipose tissue, and inflammation markers (IL-6, oxLDL, sCD14, and LBP) increased the odds of being associated with PWH.

## 4. Discussion

In this study, we found that plasma erythritol levels were significantly higher in individuals with HIV, compared to those without HIV. Additionally, we observed positive associations between plasma erythritol levels and various metabolic parameters, including BMI, adipose tissue depots, TNFrI, HbA1c, and VLDL. These findings align with previous studies that identified erythritol as a potential biomarker for metabolic and cardiovascular health. However, our study is the first one to specifically investigate these relationships in the context of HIV status, highlighting a unique aspect of metabolic dysregulation in this population.

Although dietary erythritol was partially captured via dietary recall with the nutritional analysis software used, the listing of the amount of sugar alcohols per serving on nutrition labels is not required [12]. Therefore, although the between-group differences in dietary erythritol were insignificant in this study, a more accurate account of dietary erythritol intake that may explain between-group plasma erythritol differences is unknown, due to current nutrition label requirements and the lack thereof. Whether the increased plasma erythritol in PWH in this study is secondary to a dysregulated pathway (E.g., PPP), higher dietary erythritol exposure, or another mechanism (E.g. erythritol clearance disruption, antiretroviral therapy, etc.) is presently unclear and deserves further investigations.

It has been theorized that a diet rich in glucose and/or fructose may influence endogenous erythritol production by increasing carbon flux via PPP [13]; however, our findings oppose the aforementioned theory, as the intake of total carbohydrates and total sugars were not different between PWH and controls, nor were they associated with plasma erythritol levels.

Interestingly, our study found that total dietary fiber and soluble fiber intake were significantly higher in PWoH, compared to PWH. Substantial evidence exists indicating that dietary fiber intake is inversely associated with cardiovascular disease risk, suggesting that individuals with higher fiber intake may have a reduced risk of cardiovascular events [14,15]. Despite these associations, our analysis did not reveal a statistically significant relationship between total dietary fiber or soluble fiber intake with plasma erythritol levels in unadjusted or adjusted models. This discrepancy may indicate that while dietary fiber has well-documented cardiovascular benefits, its influence on plasma erythritol levels is minimal or possibly mediated by other factors not accounted for in our study.

While several studies have highlighted the potential benefits of exogenous erythritol, caution is warranted, as several studies have linked erythritol to cardiovascular and immunologic derangements [1].

Several articles have summarized the possible beneficial effects of exogenous erythritol on health, stating that acute doses of erythritol do not affect blood levels of glucose or insulin [13,16,17]. Moreover, patients with diabetes who consumed erythritol in an interventional trial exhibited a significant decrease in hemoglobin A1C (HbA1c) after two weeks, while their blood glucose levels remained unchanged [18].

Conversely, erythritol has been associated with several factors that may negatively affect cardiovascular health. Hootman et al. identified a correlation between erythritol levels and central adiposity changes, suggesting that erythritol metabolism could be linked to metabolic health outcomes. Higher baseline erythritol levels were associated with increased central adiposity, implicating erythritol’s potential involvement in metabolic pathways that affect body weight and fat distribution [3]. Our study also found a positive association between VAT and erythritol levels in PWH. In addition to elevated plasma erythritol levels being associated with MACE, Witkowski et al. also found increased plasma erythritol to be associated with increased platelet reactivity and thrombosis [1]. In addition, Alamri et al. evaluated the effect of exogenous erythritol on inflammation and demonstrated that an increased level of erythritol can mediate the activation of M1 macrophages, a pro-inflammatory phenotype, including increased TNF-α production and the suppression of anti-inflammatory M3 macrophages, supporting the influence of erythritol on the inflammation that may drive cardio-metabolic risks [19]. In our study, we found interesting associations between erythritol levels and inflammatory markers, specifically TNF receptors I and II, in line with prior observations in populations without HIV.

In our study, PWH exhibited higher levels of plasma erythritol, compared to PWoH. Together with previous studies that have linked higher erythritol levels to various metabolic and cardiovascular diseases, these findings raise a significant concern for PWH, considering their increased susceptibility to cardiovascular mortality and morbidity due to chronic inflammation and the use of antiretroviral medications [20,21].

In addition to assessing body composition and inflammatory markers, we evaluated endothelial function and arterial stiffness. Despite thorough analysis, no statistically significant differences were observed between PWH and PWoH in these measurements. Furthermore, no association was found between these cardiovascular markers and erythritol levels. These findings suggest that future research might benefit from utilizing more specific markers to assess atherosclerotic disease in this population to better understand a possible correlation.

### Limitations

This study has several limitations that should be acknowledged. First, we did not use matched participants between groups. Consequently, the participants with HIV were significantly older, had higher body mass index, and were more likely to be non-white and male. While we adjusted for these differences in our statistical models, residual confounding may still exist, and the adjustments may not fully account for all potential interactions among variables.

Second, we relied on 3-day dietary recalls to assess the participant’s dietary intake and to quantify dietary erythritol to compare against plasma erythritol. These recalls did not immediately precede blood sample collection. To this end, it is possible that food and beverage items consumed immediately prior to blood sampling may have contained varying amounts of erythritol not captured in the diet recalls. This issue was compounded by the lack of mandatory labeling of erythritol on food labels. This limitation may lead to underreporting or misreporting of erythritol consumption, thereby affecting the reliability of our dietary intake data.

Third, the study evaluated plasma erythritol levels from participants of a parent study with exclusion criteria that included individuals with a viral load exceeding 400 copies. Consequently, it remains unclear how uncontrolled HIV, characterized by higher viral loads, might influence plasma erythritol levels, if at all. However, the current success of HIV treatment makes our population (those virologically controlled) the most pertinent in this era. These limitations highlight the need for future research to address these gaps by including matched control groups, using more comprehensive dietary assessment methods, and including participants with a broader range of HIV disease stages.

## 5. Conclusions

Our study was the first, to our knowledge, to evaluate whether plasma erythritol levels are elevated among PWH. We found positive associations between plasma erythritol and HIV status, BMI, adipose tissue, TNFrI, HbA1c, and VLDL. Despite capturing dietary erythritol intake via dietary recall, the lack of mandatory nutrition label disclosures for sugar alcohols may have led to an underestimation of dietary erythritol intake. Consequently, the precise reasons for between-group differences in plasma erythritol levels remain unclear, potentially influenced by dietary intake and or dysregulated metabolic pathways, including the pentose phosphate pathway.

Interestingly, while total dietary fiber and soluble fiber intake were significantly higher in individuals without HIV, these did not correlate significantly with plasma erythritol levels. This suggests that the well-documented cardiovascular benefits of dietary fiber do not extend to influencing plasma erythritol levels. Moreover, previous studies have highlighted both the potential benefits and risks of erythritol, including its association with increased central adiposity and major adverse cardiovascular events. Given the higher erythritol levels observed in PWH, further research is needed to explore the implications of elevated erythritol in this population, particularly considering their heightened susceptibility to cardiovascular and metabolic diseases.

## Figures and Tables

**Table 1:** Baseline characteristics by HIV status.

	HIV− (n = 53)	HIV+ (n = 109)	*p*-Value
	n (%), Median (IQR), or Mean ± std		
Characteristics			
Age (years)	44.1 ± 15.2	49.2 ± 12.2	0.01
Female Sex	31 (58.5)	46 (42.2)	0.05
Non-white Race *	14 (26.4)	71 (65.1)	<0.0001
Current Smoker (Yes)	12 (22.6)	39 (35.8)	0.09
HbA1c	5.4 ± 0.4	5.5 ± 0.5	0.03
HDL (mg/dL)	53.0 (45.8, 64.8)	45.1 (37.5, 55.0)	0.001
Non-HDL (mg/dL)	120.2 (99.7, 156.4)	133.2 (108.1, 161.5)	0.3
VLDL (mg/dL)	24 (14, 30)	23.5 (17, 36)	0.12
Triglycerides (mg/dL)	118 (71, 151)	121 (88, 181)	0.06
Cardiovascular Measures			
Pulse Wave Velocity	8.4 ± 0.9	8.6 ± 2.1	0.8
Reactive Hyperemic Index	2 (1.6, 2.3)	1.8 (1.5, 2.2)	0.2
Augmentation Index	5 (−9, 15)	4 (−4, 14)	0.8
Body Composition			
BMI (kg/m^2^)	29.4 (25.7, 33.6)	33.6 (29.1, 39.1)	0.0002
Visceral Adipose Tissue	100.7 (66.8, 134.8)	113.2 (82.3, 144)	0.1
Trunk Fat (g)	14,474.3 (10,850.7, 19,718.2)	19,501.8 (14,880.9, 24,882.1)	<0.0001
Total Fat (g)	30,026.3 (25,727.7, 38,644.8)	36,570.9 (29,280, 46,519.1)	0.003
Total LBM (g)	49,797.5 (41,905.3, 62,251.7)	59,166.7 (49,346.9, 66,482.3)	0.01
Inflammation Markers			
IL-6 (pg/mL)	2.2 (1.5, 3.2)	2.7 (1.8, 4.3)	0.02
VCAM (ng/mL)	644.4 (562.9, 775.1)	718.8 (634.1, 852.8)	0.05
TNF-RI (pg/mL)	963.20 (740.39, 1144.79)	913.9 (707, 1157.4)	0.8
TNF-RII (pg/mL)	2021.9 (1619.5, 2490.2)	2378.2 (1989, 2887.1)	0.01
hsCRP (ng/mL)	2004.7 (856.6, 5637.8)	3947.5 (1930, 7413.2)	0.01
I-CAM (ng/mL)	206.3 (165.7, 251.9)	244.4 (202.3, 287.1)	0.01
IP10 (pg/mL)	114.4 (77.9, 144.9)	154.2 (113.3, 240.3)	<0.0001
D-dimer (mg/mL)	377.71 (236, 641)	352.7 (213.3, 510.7)	0.6
oxLDL	50,576.9 (37,581.5, 69,007.3)	57,852.1 (45,675, 78,951.8)	0.03
sCD14 (ng/mL)	1550.3 (1302.2, 1745.6)	1745.1 (1503, 2129.9)	0.0001
CD163 (ng/mL)	516.8 (387.4, 694.8)	608.8 (469.1, 811.8)	0.02
Gut Markers			
Zonulin (mg/mL)	3393.5 (1829.9, 5152.9)	2906.2 (1437.1, 7859.5)	0.6
IFABP (pg/mL)	1715.9 (1315.8, 2224.4)	1902 (1275.3, 2437.4)	0.4
LBP (ng/mL)	9.7 (9.4, 10)	9.9 (9.6, 10.2)	0.01
BDG (pg/mL)	352.5 (143.1, 427.6)	211.9 (132.7, 358.7)	0.2

* Includes African American, Asian, Hispanic, and Other. Abbreviations: HDL = high-density lipoprotein; VLDL = very low-density lipoprotein; IL-6 = interleukin-6; VCAM = vascular cell adhesion molecule-1; TNF-RI = tumor necrosis factor receptor-1; TNF-RII = tumor necrosis factor receptor-2; hsCRP = high-sensitivity C-reactive protein; I-CAM = intercellular adhesion molecule-1; IFABP = intestinal fatty acid binding protein; IP10 = interferon-gamma-inducible protein of 10-kDa; oxLDL = oxidized LDL; sCD14 = soluble CD14.

**Table 2:** Baseline dietary intake by HIV status.

	HIV−	HIV+	*p*-Value
	Median (IQR), or Mean ± std		
Dietary Intake			
Total Fat (g)	65.6 (54.8, 88.4)	68.8 (49.6, 96.2)	0.9
Total Carbohydrates (g)	189.1 (141.0, 240.5)	186.2 (124.0, 241.2)	0.4
Total Protein (g)	69.8 (56.0, 86.5)	63.7 (45.6, 85.5)	0.1
Total kCal	1726.3 (1423.3, 2111.5)	1712.9 (1179.4, 2090.5)	0.4
Total Saturated Fat (g)	14.5 (10.3, 19.4)	11.7 (8.1, 15.2)	0.02
Total Monounsaturated Fat (g)	15.5 (10.3, 19.4)	12.8 (8.4, 17.2)	0.04
Total Polyunsaturated Fat (g)	8.9 (7.3, 12.4)	8.3 (4.6, 11.3)	0.1
Total Fiber (g)	13.7 (10.3, 17.5)	10.7 (7.6, 16.2)	0.05
Soluble Fiber (g)	5 (3.2, 6.4)	3.7 (2.5, 5.5)	0.02
Insoluble Fiber (g)	8.1 (5.7, 11.6)	6.6 (4.7, 10.6)	0.2
Total Sugars (g)	77.3 (45.4, 109.1)	76.7 (41.6, 113.2)	0.8
Total Erythritol (g)	0.4 ± 2.1	0.02 ± 0.3	0.5

**Table 3:** Associations with plasma erythritol.

	Unadjusted		Adjusted	
	Estimate (95% CIs)	*p*-Value	Estimate (95% CIs)	*p*-Value
HIV Status		0.03		0.001
HIV+	1.2 (1.1, 1.3)		1.2 (1.1, 1.2)	
HIV−	1.1 (1.0, 1.2)		1.1 (1.0, 1.2)	
Age	0.3 (0.1, 0.5)	0.002	0.1 (0.003, 0.16)	0.04
Sex (Female vs. Male)	−0.09 (−0.2. 0.02)	0.09	−0.1 (−0.2, 0.01)	0.03
Race (non-White vs. white)	−0.04 (−0.17, 0.08)	0.5	−0.05 (−0.2, 0.07)	0.09
BMI	0.38 (0.1, 0.68)	0.01	0.01 (0.01, 0.02)	0.001
HgbA1c	0.9 (0.3, 1.6)	0.01	0.8 (−0.4, 2.1)	0.2
VLDL	0.2 (0.1, 0.3)	0.003	0.3 (0.1, 0.5)	0.01
Visceral Adipose Tissue	0.2 (0.05, 0.4)	0.01	0.4 (0.3, 0.6)	<0.0001
Pulse Wave Velocity	0.3 (0.03, 0.6)	0.03	0.4 (−0.1, 0.9)	0.2
IL-6	0.1 (0.02, 0.2)	0.02	0.06 (−0.09, 0.2)	0.5
TNFrI	0.2 (0.1, 0.4)	0.003	0.3 (0.1, 0.6)	0.01
TNFrII	0.2 (0.1, 0.4)	0.004	0.3 (−0.01, 0.6)	0.07
iFAB-P	0.1 (0.03, 0.2)	0.01	0.1 (−0.06, 0.3)	0.2
sCD163	0.2 (0.03, 0.3)	0.02	0.2 (−0.06, 0.4)	0.1

Abbreviations: HgbA1c = hemoglobin A1c; TNFrI = tumor necrosis factor receptor-1; TNFrII = tumor necrosis factor receptor-2; iFAB-P = intestinal fatty acid binding protein; sCD163 = soluble CD163.

**Table 4:** Risk factors associated with HIV status.

	uOR (95% CI); *p*-Value *	aOR (95% CI); *p*-Value **
Erythritol [μM] Categories		
2.8–3.5 vs. <2.7 [μM]	2.3 (0.9, 5.4); *p* = 0.07	2.4 (0.9, 6.1); *p* = 0.06
>3.6 vs. <2.7 [μM]	2.3 (1.1, 4.9); *p* = 0.03	2.5 (1.1, 5.5); *p* = 0.03
Erythritol [μM] ***	2.8 (1.1, 7.5); *p* = 0.03	2.8 (1.1, 8.2); *p* = 0.04
Subcutaneous Adipose Tissue ****	2.7 (1.2, 5.9); *p* = 0.01	2.01 (0.9, 4.6); *p* = 0.1
IL-6 (pg/mL)	1.8 (1.1, 3.1); *p* = 0.02	1.5 (0.9, 2.6); *p* = 0.1
oxLDL	2.4 (1.1, 5.1); *p* = 0.03	1.7 (0.8, 4.04); *p* = 0.2
sCD14 (ng/mL)	1.002 (1.001, 1.003); *p* = 0.0001	1.002 (1.001, 1.003); *p* = 0004
LBP (ng/mL)	3.2 (1.5, 6.8); *p* = 0.002	1.001 (1.001, 1.001); *p* = 0.01

* uOR = unadjusted odds ratio; CI = confidence interval. ** aOR = adjusted odds ratio. *** Erythritol = continuous. **** BMI was not included with subcutaneous adipose tissue in adjusted models.

## Data Availability

Data produced under this proposal will be made available to investigators upon request to the corresponding author after research completion and publication for those with institutional review board-approved proposals and signed university-approved material and data transfer agreements. Individual participant data will be shared in data sets in a de-identified and anonymized format.
